# Interaction of mRNA with the C-Terminal Domain of PCID2, a Subunit of the TREX-2 Complex, Is Required for Its Export from the Nucleus to the Cytoplasm in *Drosophila melanogaster*

**DOI:** 10.1134/S1607672923700527

**Published:** 2023-12-08

**Authors:** Yu. A. Vdovina, S. G. Georgieva, D. V. Kopytova

**Affiliations:** Engelhardt Institute of Molecular Biology, Russian Academy of Sciences, Moscow, Russia

**Keywords:** TREX-2, PCID2, mRNA export, transcription

## Abstract

Following the transcription step, the newly synthesized mRNA is exported from the nucleus to the cytoplasm and further to the translation site. The TREX-2 complex is involved in the step of mRNA export from the nucleus to the cytoplasm. This complex in *Drosophila melanogaster* consists of four proteins: Xmas-2, PCID2, ENY2, and Sem1p. In our work, we have shown that deletion of the C-terminal sequence of PCID2 leads to a decrease in the interaction of the protein with RNA and to impaired mRNA export from the nucleus to the cytoplasm in *D. melanogaster*.

The process of gene expression consists of many stages, at each of which it is regulated by certain protein complexes. The TREX-2 complex, which is involved in the export of mRNA from the nucleus, in *D. melanogaster* consists of four proteins: Xmas-2, PCID2, ENY2, and Sem1p [1–3]. Some of the subunits of this complex are also involved in other stages of gene expression. It is known that one of the subunits of the complex, the ENY2 protein, is also a component of the SAGA complex and interacts with the THO complex, thus participating in both the transcription initiation and elongation stages [4–6]. dENY2 is also involved in the barrier activity of various insulators [7, 8]. The PCID2 protein participates not only in the export of mRNA in the nucleus, but also in the transport of mRNA in the cytoplasm [9, 10]. In the complex, PCID2 interacts with mRNA in RNA immunoprecipitation reactions and, possibly, determines the specificity of the interaction of TREX-2 with mRNA [10]. Although the functions of the TREX-2 complex have been studied for a long period of time, it remains unclear how the complex interacts with mRNA [11–17]. Studies in yeast showed that Xmas-2 and PCID2 homologs together bind RNA to form a common binding surface [18]. In the yeast PCID2 homolog, the domain located at the C terminus of the protein is responsible for binding to RNA. The amino acids involved in the interaction have been identified [18]. In our work, we studied whether this domain is involved in binding to RNA in *D. melanogaster* PCID2 and whether this domain is required for performing the key functions of PCID2. The interaction of PCID2 with mRNA in *D. melanogaster* was studied using the biotinylated RNA pull-down technique.

Biotinylated mRNA of the *ras2* gene was used as a model RNA the binding to which was studied, since the interaction of PCID2 in the complex with this mRNA was shown previously in RNA immunoprecipitation reactions [10]. In the experiment, we used the following proteins expressed in the bacterial system: the full-length PCID2 and PCID2^1–360^ (PCID2 in which 35 amino acid residues at the C-terminus of the protein were removed). The scheme of proteins is shown in Fig. 1a.

**Fig. 1.**
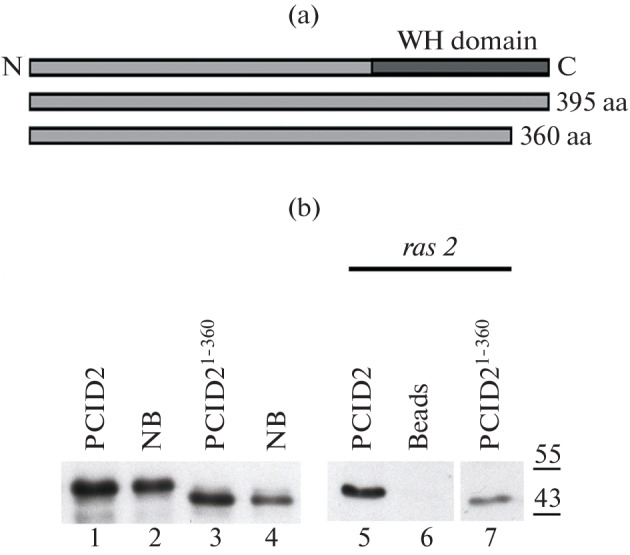
C-terminal domain of PCID2 interacts with *ras2* mRNA in pull-down experiments. (a) Domain structure of the PCID2 protein. The figure shows the full-length protein and PCID2^1–360^, in which 35 amino acid residues at the C-terminus were removed. (b) Western blot analysis of protein binding to biotinylated *ras2* mRNA. The membrane was hybridized with antibodies to PCID2 [1]. Lanes: 1, 3—expressed proteins taken for binding; 2, 4—NB (fractions of proteins not bound to *ras2* mRNA); 5, 7—fractions containing proteins bound to *ras2* mRNA; 6—fraction that ensured the binding of *ras2* mRNA to streptavidin–agarose.

Proteins were bound to biotinylated *ras2* mRNA, incubated with streptavidin–agarose, and washed from the unbound protein with a high-salt solution. The protein bound to mRNA was visualized by PAGE separation and Western blot hybridization with antibodies to PCID2 (Fig. 1b). It turned out that the PCID2^1–360^ form binds to *ras2* mRNA much less efficiently than the full-length PCID2. Thus, the RNA-binding domain in *D. melanogaster* PCID2 is located at the C-terminus of the protein, in those 35 amino acids that were removed. However, since the protein binding to *ras2* RNA did not disappear completely, it can be assumed that PCID2 also has another RNA-binding domain.

The effect of this deletion on the main function of PCID2—the export of mRNA from the nucleus to the cytoplasm—was studied in *D. melanogaster* S2 cells. PCID2 was knocked down in the cells using RNA interference, and cells were transfected with the pAC-PCID2^1–360^ construct with the HA epitope. The cells with PCID2 knockdown that were transfected with the pAC-PCID2 construct with the HA epitope were used a control (Figs. 2a, 2b).

**Fig. 2.**
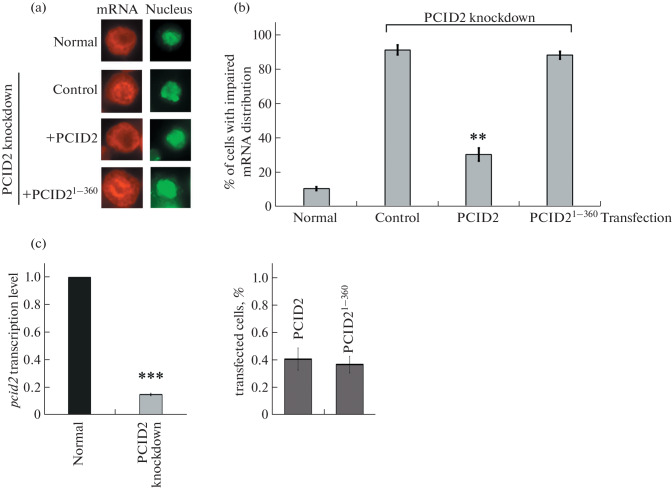
C-terminal domain of PCID2 is required for mRNA export. (a) FISH RNA was performed using a Cy3-labeled oligo-dT primer to visualize polyA RNA, and nuclei were stained with DAPI. Cells were treated with double-stranded RNA corresponding to PCID2. PCID2-knockdown cells were used in the experiment as a positive control. Distribution of mRNA (green signal) and cell nucleus (red signal) are shown for untreated cells (normal), cells with PCID2 knockdown (control), PCID2-knockdown cells transfected with the full-length PCID2 (+PCID2), and PCID2-knockdown cells transfected with PCID2^1–360^ (+PCID2^1–360^) (1000× magnification). (b) Quantitative representation of the results of the experimental shown in (а). Histograms show the percentage of cells with impaired mRNA export (approximately 200 cells were examined). (c) Histograms show the level of *pcid2* mRNA interference and the level of transfection of interfered S2 cells with PCID2 and PCID2^1–360^.

The level of protein knockdown was analyzed using Western blot hybridization; the percentage of transfected cells was determined by the number of cells immunostained with antibodies to the HA epitope, presented in the form of histograms (Fig. 2c). In normal, untreated cells, mRNA was distributed unevenly, predominantly in the cytoplasm. PCID2 knockdown resulted in mRNA retention in the nucleus and redistribution in 91% of cases. Transfection with the full-length protein against the background of knockdown led to a decrease in the number of aberrant cells by up to 27%. At the same time, transfection with the PCID2^1–360^ protein had no such an effect, and the number of cells with retention and redistribution of mRNA did not change (88%). The results of this experiment suggest that the C-terminal domain is required for PCID2 functions in the nucleus.

Thus, in this study, we showed that the C-terminal domain of *D. melanogaster* PCID2 is responsible for the interaction of the protein with RNA and is required for efficient export of mRNA from the nucleus to the cytoplasm. The presented data also showed that, unlike the yeast PCID2 homolog, *D. melanogaster* PCID2 has also other RNA-binding domain(s).
